# Short-duration podcasts as a supplementary learning tool: perceptions of medical students and impact on assessment performance

**DOI:** 10.1186/s12909-017-1001-5

**Published:** 2017-09-18

**Authors:** S.S. Prakash, N. Muthuraman, R. Anand

**Affiliations:** Department of Biochemistry, Christian Medical College, Vellore, Tamil Nadu 632002 India

**Keywords:** Undergraduate Medical Education, Webcasts, Podcasts, Biochemistry, Medical students

## Abstract

**Background:**

Use of podcasts has several advantages in medical education. Podcasts can be of different types based on their length: short (1–5 min), moderate (6–15 min) and long (>15 min) duration. Short-duration podcasts are unique since they can deliver high-yield information in a short time. The perceptions of medical students towards short-duration podcasts are not well understood and this study aimed to analyze the same. An exploratory analysis of students’ podcast usage and performance in summative assessments was also undertaken.

**Methods:**

First-year medical students (*N* = 94) participated in the study. Eight audiovisual podcasts, each ≤3 min duration (3-*M*inu*T*e *L*essons; 3MTLs) were developed for two topics in biochemistry. The podcasts were made available for students after didactic lectures on the topics. Feedback was collected from students about their perceptions to 3MTLs using a self-reported questionnaire. The scores of students in summative assessments were compared based on their usage of 3MTLs.

**Results:**

Feedback revealed that 3MTLs were well received by students as a useful and convenient supplementary tool. Students used 3MTLs for topic review, to get an overview, as well as for quick revision and felt that 3MTLs were helpful in improving their understanding of the topic, clarify concepts and focus on important points and in turn, in preparation for assessments. A significant proportion (49%) felt that 3-min duration was optimal while, an equal proportion suggested an increase in the duration to 5 min with more information. The overall mean scores in assessments were not different between students based on 3MTLs usage. The pairwise comparisons revealed better scores amongst students who used 3MTLs for both topics.

**Conclusion:**

Overall, short-duration podcasts were perceived by students as useful supplementary learning tools that aided them for revision and in preparation for assessments.

**Electronic supplementary material:**

The online version of this article (10.1186/s12909-017-1001-5) contains supplementary material, which is available to authorized users.

## Background

Biochemistry is considered by medical students as a difficult subject to study, while it’s also an equally challenging task for the teachers to teach [1]. Unlike the subjects where it may be possible for students to correlate with specimens, dissections, or real life examples, conceptualization in biochemistry is often, a challenge [1]. Traditional didactic teaching and textbooks may not suit all students since learning preferences are different among individuals [2]. The new generation of medical students is more proficient with the use of technology. Hence, educators need to come up with strategies to assist self-learning [3]. In this context, podcasts can be a useful educational technology that can cater to learners with auditory, visual or mixed learning preferences [4]. The term ‘podcast,’ now refers to audio/video files that can be downloaded or streamed on portable media players [5]. Podcasting was initially developed for entertainment but has been tried in the fields of medical, dental, nursing and veterinary education [6–9]. Understandably, students have the advantage to access podcasts by devices of their choice (smartphones, tablets, and computers). Since podcasts can be used anywhere, anytime, they enable students to learn at their pace [5, 10].

There is a notable enthusiasm among educators for the adoption of podcasts into medical education [11–13]. On the other hand, a few educators also raise caution in using podcasts, citing lack of credible evidence [14]. Additionally, the success of this technology also depends on other factors such as the purpose of podcasts, type of podcasts, mode of delivery, target audience, etc. Hence, there are still gaps in our understanding of the utility of podcasts in medical education. Despite the available evidence to support the use of podcasts, currently, there is no defined role for it in medical education [14]. The consensus is that podcasting should be used to complement rather than replace traditional teaching for a richer learning experience [6, 12, 15].

In the past, several studies have used podcasts in medical education for different purposes. Podcasts have been tried as a teaching method in comparison to traditional didactic teaching [8, 16–21] or text-based learning [22, 23]. Most other investigators have used podcasts as tools to supplement routine teaching practices [6, 7, 10, 24–34]. Alternatively, a few investigators have tried podcasts as a preparatory tool for “flipped-classroom” which allows teaching hours to be utilized for problem-solving interactions [35, 36].

An additional factor that can influence the success of podcasts, albeit less studied, is the length of podcasts. Based on a classification by Carvalho et al., podcasts can be of different types based on their length: short (1–5 min), moderate (6–15 min) and long (>15 min) duration [37]. A common practice among several universities has been to record and provide entire lecture sessions, analogous to long-duration podcasts [38–40]. However, it is known that long duration podcasts are less preferred by listeners [41]. The general recommendation is to maintain the length of podcasts <15 min, considering the attention span of listeners [37, 38, 42]. In the past, several researchers have mainly used moderate-duration podcasts for their investigation [6, 8, 27, 29, 30, 34]. Short-duration podcasts are less studied, and there are only a few studies in this regard [28, 43, 44]. Aguiar et al. used short-duration podcasts for delivering learning outcomes and guidelines for reading the topic without providing any conceptual information, as such [43]. In a study by Narula et al.*,* clinical clerks were provided with 5-min podcasts each designed to cover a selected core internal medicine concept [28]. Another study used short-duration podcasts for information delivery and student feedback on evolution and heredity topics [44]. However, students’ perceptions towards short-duration podcasts are less understood.

Short-duration podcasts are unique since they are capsules of high-yield information for students to review quickly. However, an inherent disadvantage of short-duration podcasts is the limited information that can be conveyed due to the time barrier. This factor can be critical from the students’ perspective; because gathering information within short-duration may require some prior knowledge on the topic. Hence, it is important to analyze the usefulness of podcasts among medical students. This pilot study was undertaken to analyze the perceptions and preferences of medical students towards short-duration podcasts in biochemistry. An exploratory analysis of students’ usage of podcasts and its impact on performance in summative assessments was also undertaken.

## Methods

The study was conducted in the Department of Biochemistry at Christian Medical College, Vellore, India from December 2015 to March 2016. The study protocol was approved by the Institutional Review Board (IRB). First-year medical students (*N* = 100) from the Batch of 2015 from the institution were invited to participate in the study. Since the investigator (AR) and participants were acquainted with each other, the study had a post-positivistic approach. The investigator’s speculation was that short-duration podcasts would be useful as supplementary learning tools.

### Podcast development and delivery

A decision on the type of podcast to be used for this study was based on the taxonomy proposed earlier [37]. Accordingly, the type of podcasts was *informative*; the medium was *videos* of *short-duration* (≤ 3 min per episode), the author was the *investigator* (AR) himself, the style was *formal,* and the purpose was *to inform*. All podcasts (3-*M*inu*T*e *L*essons; 3MTLs) consisted of a graphical slide show with a video inset of the narrator in each slide. The slide show was created using Microsoft PowerPoint™ (Microsoft, WA, USA) and audio-visual component was recorded using Office Mix™ (Microsoft, WA, USA). The script for the podcasts was written in advance, tailored to fit the time-frame and was read out by the investigator during the recording.

Initially, a pilot episode was recorded by the investigator. Faculty members (*n* = 5), postgraduate students (*n* = 3) and second-year medical students (*n* = 3) from the institution were invited to review the pilot podcast and provide an informal feedback (data not shown). Subsequently, eight 3MTLs were developed for two topics taught under biochemistry curriculum (Table 1). The content of 3MTLs was designed in such a way that only brief highlights of the topics were covered. All 3MTLs used in this study had 320 × 240 image resolution with file sizes between 6.2 to 8.8 MB such that they would be optimal for use with small screen devices without occupying a lot of storage space. Production of each 3MTL required around 6–8 h inclusive of planning, rehearsal sessions, and final recording.

**Table 1:** List of podcast episodes and learning objectives

S. No.	Podcast topic	Duration	Specific learning objectives	Total views^a^	Total visitors^a^
3MTLs – Fat soluble vitamins					
1.	Vitamin A	3′ 00ʼ	a) Name the active forms of vitamin A and their physiological role b) List the dietary sources of vitamin A c) List the functions of vitamin A d) List the clinical features of vitamin A deficiency	91	51
2.	Vitamin D: metabolism and functions	2′ 55ʼ	a) Name the active form of vitamin D b) Define the role of skin, liver and kidneys in the metabolism of vitamin D c) List the functions of vitamin D	69	45
3.	Regulation of vitamin D metabolism and its deficiency	2′ 30ʼ	a) List the factors regulating vitamin D metabolism b) List the causes for vitamin D deficiency c) List the clinical manifestations of vitamin D deficiency	44	34
4.	Vitamin K and Vitamin E	2′ 53ʼ	a) Name the active forms of vitamin K and E b) Explain the biological roles of vitamin K and vitamin E c) List the proteins activated by vitamin K d) Explain the effect of warfarin on vitamin K metabolism e) List the clinical manifestations of vitamin K deficiency	46	34
3MTLs – Heme metabolism and disorders of hemoglobin					
5.	Heme biosynthesis	2′ 57ʼ	a) List the precursors for heme biosynthesis b) Name the major tissues involved in heme biosynthesis c) Name the rate limiting enzyme for heme biosynthesis d) Outline the biosynthesis of heme	90	44
6.	Porphyrias	2′ 59ʼ	a) Classify porphyrias b) List the common clinical features of porphyrias c) Explain the basis behind clinical presentation of porphyrias	68	33
7.	Disorders of hemoglobin – Sickle cell disease	2′ 56ʼ	a) Explain the molecular defect in sickle cell disease b) Explain the biochemical basis of symptoms in sickle cell disease	68	34
8.	Disorders of hemoglobin - Thalassemia	2′ 52ʼ	a) Explain the molecular defect in thalassemia b) Explain the biochemical basis of symptoms in thalassemia	47	33

^a^Data obtained from the institution’s e-learning portal (http://e-learning.cmcvellore.ac.in) as on 27^th^ Feb 2016

The topics were initially delivered as lectures of 1-h duration each, by the investigator (AR) on eight different occasions (Table 1). Subsequent to each lecture, 3MTL on the topic was made available on the institution’s e-learning portal (http://e-learning.cmcvellore.ac.in). This education portal was developed in collaboration with Tufts University School of Medicine, Boston, Massachusetts, USA, on an open source platform called the Tufts University Sciences Knowledge Base (TUSK) (for more details on the portal, refer to [45, 46]). After the upload of the first 3MTL in the series, students were announced about the availability of 3MTLs in the e-learning portal and mode of access. Students were allowed to stream and download 3MTLs without restrictions. As per routine practice, lecture slides were also made available for the students. Written assessments were conducted as part of the students’ ongoing schedule. Cumulative data on students’ usage was monitored using a tracking option available in the e-learning portal.

### Survey

Feedback was collected in a classroom setting from the students who provided informed consent. A two-part questionnaire developed for this study was used for data collection. The questionnaire was reviewed for construct validity by two faculty members and two postgraduate students not involved in the study. Part A questionnaire was designed to obtain anonymous feedback and suggestions from students and contained two open-ended items (A1 and A2). Part B questionnaire (Additional file 1) was designed to collect specific data with closed-ended items (B1-B16). Items B1 to B8 were questions about the educational background of students (B1-B4) and their study habits (B5-B8). Items B9-B13 were designed to assess the pattern of usage of 3MTLs by the students. A 5-point Likert scale was used for B14 (where, 1 = poor, 2 = fair, 3 = good, 4 = very good and 5 = excellent) and B15 (where, 1 = not useful, 2 = somewhat useful, 3 = useful, 4 = very useful and 5 = extremely useful) to assess the quality of 3MTLs and their usefulness from the students’ perspective, respectively. The aspects evaluated in B14 were *clarity of presentation, use of simple, clear language, adequacy of content, quality of audio, quality of video and ease of downloading (streaming) on e-learning*. Item B16 asked students if they would recommend 3MTLs for other topics in the future. Data was extracted from the completed questionnaires and participants were anonymized for analysis.

### Qualitative analysis

Qualitative data analysis for responses from items A1 and A2 were carried out using a previously published coding scheme after adaptation [47]. The qualitative analysis of both items was scored on 5-point rating scale under three major categories: *learning, engagement, and quality*, each of which was further subcategorized. The coding scheme used for analysis is shown in Table 2. All responses by students were rated for each subcategory by two raters independently (SSP and MN) and discrepancies were resolved by consensus. The average rating was calculated for each subcategory, and total effect of each was calculated by multiplying the average rating by the total number of students who commented on the respective subcategory.

**Table 2:** Coding scheme for qualitative analysis of items A1 and A2

Category label	Criteria
A1. Did you find the 3-min videos helpful? Please elaborate	
Learning	
• Challenge	Student refers to the ease/difficulty of concepts covered in 3MTLs
• Learn	Student refers to a general learning aspect about 3MTLs
• Revision	Student refers to a help/lack of thereof provided by 3MTLs for revision
• Overview	Student refers to a help/lack of thereof provided by 3MTLs for topic overview
• Study	Student refers to a help/lack of thereof provided by 3MTLs for studying the topic
• Audio	Student refers to audio feature in 3MTLs that helped/inhibited learning
• Visual	Student refers to visual feature in 3MTLs that helped/inhibited learning
• Time	Student refers to a comment about time being a factor in 3MTLs
• Help	Student refers specifically to help/lack of thereof provided by 3MTLs towards preparation for assessments
Engagement	
• Compare	Student compares use of 3MTLs to another method of learning
• Engage	Student refers to 3MTLs as being OR not being fun/enjoyable/engaging/interesting
• Technology	Student mentions a technological issue with respect to using 3MTLs
Quality	
• Ease	Student refers to ease/difficulty with using 3MTLs
• Control	Student refers to control of choice/pace in using 3MTLs
• Graphics	Student refers to a comment about picture or animations in 3MTLs
• Text	Student refers to a comment about text in 3MTLs
• Language	Student refers to a comment about ease/difficulty about language used in 3MTLs
*Rating scale: −2 = Very negative comment; −1 = Negative comment; 0 = Neutral or no mention; 1 = Positive comment; 2 = Very positive comment*	
A2. Do you have any suggestions to improve the videos?	
Learning	
• Challenge	Student suggests about improvement related to concepts covered in 3MTLs
• Learn	Student suggests about improvement related to a learning aspect about 3MTLs
• Time	Student suggests a comment about increasing/decreasing time of 3MTLs
• Number	Student suggests a comment about increasing/decreasing the number of 3MTLs
Engagement	
• Compare	Student suggests improvement in 3MTLs in comparison to another method of learning
• Engage	Student suggestion refers to improving engagement of audience in refers to 3MTLs (being fun/enjoyable/engaging/interesting)
• Technology	Student suggests improvement with a technological issue with respect to using 3MTLs
Quality	
• Control	Student suggests about improvement to control of choice/pace in using 3MTLs
• Graphics	Student suggests improvement about picture or animations in 3MTLs
• Text	Student suggests improvement about text in 3MTLs
• Language	Student suggests improvement about ease/difficulty about language used in 3MTLs
• Audio	Student suggests improvement about audio feature in 3MTLs
• Visual	Student suggests improvement about visual feature in 3MTLs
• Clarity	Student suggests improvement about clarity of presentation in 3MTLs
*Rating scale for A2: −2 = Needs major improvement; −1 = Needs minor improvement; 0 = Neutral or no mention; 1 = Just adequate at present; 2 = No further improvement necessary*	

### Quantitative analysis

Students were divided into 3 groups based on self-reported 3MTL usage [3MTL: Heavy-users (used 3MTLs for two topics); 3MTL: Intermediate-users (used 3MTLs for one topic); 3MTL: Non-users (did not use 3MTLs)]. Assessment scores of students were obtained from departmental records. Data integrity was verified by random cross checking at two different time points. The impact of 3MTL usage was studied by comparing scores obtained by students in written assessments. Each assessment constituted a written test for a maximum score of 30 and absolute scores obtained by students were used for quantitative analysis. The assessment questions were essay and short answer descriptive type, testing a combination of factual recall and application-based knowledge. Faculty members in the department (including investigators on a rotational basis, as per departmental policy) were involved in the design of questions for assessment and scoring the answer scripts. Questions used in assessments were screened for construct validity by a faculty member not part of the study.

Test_3MTL_ denotes the assessment conducted on topics in Table 1 (which had 3MTL supplementation). Test_P_ and Test_S_ refer to the assessments that immediately preceded and succeeded Test_3MTL_, respectively. Scores of students in 5 historic assessments (conducted before Test_P_) were also obtained. Although each of the assessments covered different modules (Additional file 5: Table S4), the pattern of assessments was similar. Only topics covered for Test_3MTL_ had podcast supplementation; also, content for Test_3MTL_ as well as other assessments was based on curricular requirements and not strictly on the learning outcomes of 3MTLs.

To study intra-individual changes, pairwise comparison of scores were calculated (Test_3MTL_ minus Test_P_; Test_3MTL_ minus Test_S_). For instance, if a student’s score in Test_P_, Test_3MTL_ and Test_S_ was 24, 27 and 23, respectively, the pair differences in scores would be: Test_3MTL_ minus Test_P_ = 3 and Test_3MTL_ minus Test_S_ = 4. Subsequently, an average of the pairwise differences in scores was calculated for 3MTL: Heavy-users, Intermediate-users, and Non-users. To test if a subset of students in 3MTL: Heavy-users may be the primary beneficiaries of 3MTLs accounting for the above observation, 3MTL: Heavy-users were further sub-classified into tertiles based on their average scores in historical assessments as Below-average performers, Average performers, and Above-average performers.

### Statistical analysis

Likert scale data were handled as ordinal data [48]. Parametric tests were used for analysis. ANOVA followed by Bonferroni’s posthoc test used for comparison of Test_3MTL_ scores between different groups. For pairwise comparisons (Test_3MTL_ vs Test_P_/ Test_3MTL_ vs Test_S_), paired t-test was used. All statistical analyses were done with SPSS v. 16 software. Reporting of the study is as per Standards for Reporting of Qualitative Research (SRQR) guidelines [49] considering additional recommendations related to medical education research [50–52].

## Results

Ninety-four students consented to participate in the study. Feedback indicated that 41 students (43.6%) used 3MTLs for both topics, 34 students (36.2%) used for one of the topics while 19 students (20.2%) did not use 3MTLs. The usage statistics from the e-learning portal indicated that at the end of the study period, the total numbers of views and visitors for 3MTLs were 477 and 308, respectively (Table 1).

Ninety-one students responded for Item A1 of feedback questionnaire which asked *‘Did you find the 3-Minute Lesson videos useful? Please elaborate,’* and 79 students (86.8%) mentioned that 3MTLs were useful. Summary of comments for item A1 is shown in Table 3 (for detailed comments, see Additional file 2: Table S1). Qualitative analysis for item A1 revealed that the majority of students’ comments were about learning category, specifically to help offered by 3MTLs for revision, preparation for assessments and general learning which were rated the top 3 subcategories. A few students specified 3MTLs were helpful in gaining an overview of the topic and studying. A few students also mentioned the advantage of the short-duration of the podcasts and the teacher’s voice in the audio component. In the engagement category, some students specified that 3MTLs were more useful and engaging compared to reading textbooks or lecture slides. Very few comments from students were about the quality aspects of the podcasts. Of note, the students’ ability to control the podcasts and convenience in using them were mentioned by a few students. Technological issues faced by students in using 3MTLs was rated the lowest among all subcategories. Eleven students who indicated they did not use 3MTLs, specified reasons such as, they *‘prefer reading’* and that *‘slides were easier to download.’*

**Table 3:** Qualitative analysis of responses for item A1

Categories	Total responses for A1 (*N* = 91)		No of comments (n)	Total effect (Mean x n)	Paraphrases of sample comments from students
	Mean	SD			
Learning					
• Revision	0.97	0.85	57	55.1	*‘useful for revision especially at the last-minute’ (#1, 2, 3, 4, 5, 6, 7, 8, 9, 10, 11, 12 14, 15, 16, 17, 18, 19, 21, 22, 23, 24, 27, 28, 29, 32, 35, 36, 38, 39, 40, 41, 43, 45, 46, 48, 49, 50, 51, 53, 55, 56, 57, 58, 59, 60, 61, 63, 67, 68, 69, 70, 71, 73, 75, 78, 91)*
• Help	0.53	0.83	28	14.77	*‘useful before assessments’ (#2, 4, 5, 6, 8, 15, 18, 19, 21, 22, 26, 28, 29, 33, 34, 35, 38, 40, 45, 46, 53, 61, 62, 66, 67, 68, 69, 73)*
• Learn	0.27	0.56	20	5.49	*‘made it easier to recollect points’ (#34, 63)* *‘helped me understand concepts better and clarify a few doubts’ (#11, 20, 26, 30, 58, 62, 88)* *‘covered all the main facts and summarized the whole chapter’ (#9, 44, 54)* *‘useful to have concise forms of lectures (#1, 6, 19, 55)* *‘helped in understanding the categorization and layout of specific topics’(#21, 67)* *‘helped to focus on important points and catch-up things missed by merely reading’ (#45, 57)*
• Overview	0.16	0.52	9	1.48	*‘helpful for a quick overview’ (#47, 52, 54, 65, 71, 74, 79)* *‘watching the videos before I started reading the gave me a head-start on what I’m supposed to know’ (#37, 44)*
• Time	0.11	0.38	8	0.88	*‘everything important was condensed into 3 min and doesn’t take much of time’ (#1, 17, 40, 55, 65, 68, 71, 82)*
• Study	0.1	0.4	7	0.8	*‘very helpful for last minute preparation’ (#33, 36, 45, 62, 63, 66, 70)*
• Audio	0.09	0.35	6	0.53	*‘teachers voice in the background was helpful, reassuring and gave a personal touch’ (#17, 41, 43, 45, 72)* *‘instead of listening to music sometimes you could listen to videos for recap’ (#12)*
• Challenge	0.05	0.27	4	0.22	*‘easy to understand’ (#9, 11, 51, 62)*
• Visual	0.02	0.26	3	0.07	*‘audiovisual aids can be more useful for some students’ (#43)* *‘teacher’s interaction through the video made it lively’ (#71)* *‘video could have been improved a lot’ (#83)*
Engagement					
• Compare	0.2	0.5	15	2.97	*‘easier than going through text books or lecture slides’ (#8, 42, 62, 73)* *‘helped me understand better after I have gone through the slides’/‘catch-up things missed by merely reading slides’ (#11, 45, 50, 51)* *‘can be more useful for some students than reading books’ (#2, 43, 52)* *‘useful to revise and recollect points since it was like listening through the lecture again (#17, 88)* *‘didn’t watch the videos because I prefer to study’ (#59)*
• Engage	0.07	0.36	6	0.40	*‘interesting, less boring and lively than going through the e-earning slides’ (#8, 10, 42, 58, 71)* *‘seemed like slides were just being read out, which I can do by myself’ (91)*
• Technology	−0.03	0.18	3	−0.1	*‘faced issues with downloading the files’ (#76, 85, 86)*
Quality					
• Control	0.05	0.31	3	0.16	*‘I was able to pause and listen, when and where I wanted’ (#51, 62)* *‘If I had doubt after watching the video I could read once again for clarification’) (#34)*
• Ease	0.045	0.29	2	0.09	*‘convenient to study with phones’ (#57)* *‘heard quite a few of them walking to the exam hall’ (#69)*
• Graphics	−0.01	0.10	1	−0.01	*‘animations could have been added’ (#83)*
• Text	0.00	0.00	0	0.00	–
• Language	0.00	0.00	0	0.00	–

Summary of qualitative analysis and paraphrases of their responses for items A1 are presented here (For detailed comments see Additional file 2: Table S1). Numbers in the parenthesis indicate the serial number of students’ responses

Item A2 was ‘*What suggestions do you have to improve the 3-Minute Lesson videos?’* and it received 76 responses. Summary of responses to item A2 is shown in Table 4 (for detailed comments, see Additional file 3: Table S2). In the learning category, the majority of students’ comments suggested improving the challenge component of 3MTLs by including more concepts and extending to other complex topics in biochemistry. Another suggestion was to increase the duration of podcasts. In the engagement category, the major suggestion was to address the technological issues in using 3MTLs by making them easier to download and decreasing the size of the files. A few students also suggested increasing the interactivity of podcasts. In the quality category, the suggestions were to improve the quality of audio, video, and graphics used in 3MTLs.

**Table 4:** Qualitative analysis of responses for item A2

Categories	Total responses for A2 (*N* = 76)		No of comments (n)	Total effect(Mean x n)	Paraphrases of sample comments from students
	Mean	SD			
Learning					
• Challenge	−0.26	0.44	20	−5.26	*‘expand to other bigger/complex topics’ (#7, 15, 16, 50, 57, 60, 71, 73)* *‘include more concepts’ (#4, 13, 22, 25, 29, 32, 34)* *‘would be more useful if it is more explanatory/elaborate’ (#7, 21)*
• Time	−0.1	0.5	13	−1.2	*‘longer videos with more concepts’ (#4, 21, 22, 24, 34, 41, 52, 57, 62, 67)* *‘shortness of the videos is interesting’ (#71)*
• Number	−0.08	0.32	5	−0.39	*‘make more such videos’ (#50, 54, 57, 61, 73)*
• Learn	−0.07	0.25	5	−0.33	*‘please mention assessment questions that may come from the topic’ (#27, 51, 63)* *‘stress more on important points’ (#27, 36)*
*Engagement*					
• Technology	−0.11	0.31	8	−0.84	*‘make it easier to download’ (#9, 31, 32, 33, 43, 70)* *‘make size of files smaller as they take up lot of memory’ (#43, 70)* *‘if possible upload the videos on YouTube’ (#69)*
• Engage	−0.07	0.25	5	−0.33	*‘videos could be more interactive’ (#2, 3, 55, 61)* *‘add more content for sparking interest in the topic’ (#75)*
• Compare	0.03	0.23	1	0.03	*‘play it in class too as lectures are boring’ (#54)*
*Quality*					
• Audio	−0.1	0.3	10	−1.3	*‘speak loudly and slowly’ (#1, 74)* *‘improve audio quality’ (#11, 14, 22, 31, 34, 52, 62, 76)*
• Graphics	−0.08	0.32	5	−0.39	*‘use more animations/pictures/graphics’ (#3, 5, 10, 38, 72)*
• Visual	−0.1	0.22	4	−0.2	*‘improve video quality/resolution’ (#11, 48)* *‘since speaker’s face is not that important, if avoided can save preparation time’ (#2, 26)*
• Clarity	−0.04	0.2	3	−0.12	*‘more clarity needed’ (#66, 68, 74)*
• Control	−0.03	0.16	2	−0.05	*‘pace of videos could be slower’ (#1, 31)*
• Language	−0.01	0.11	1	−0.01	*‘instead of using same sentences in the slides, use your own language and explain’(#8)*
• Text	0	0	0	0	–

Summary of qualitative analysis and paraphrases of their responses for items A2 are presented here (For detailed comments see Additional file 4: Table S3). Numbers in the parenthesis indicate the serial number of students’ responses

**Fig. 1:**
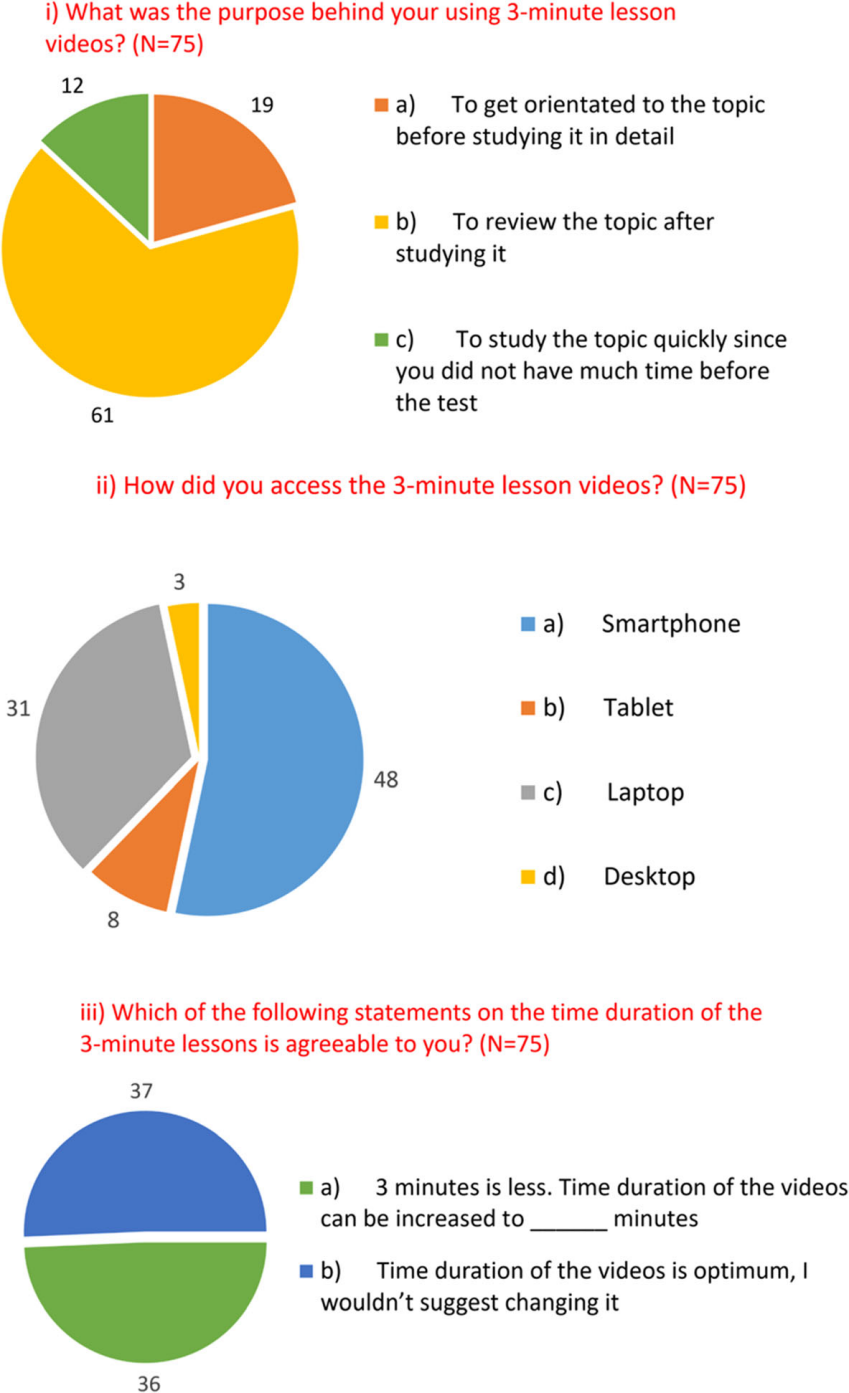
Students’ perception and mode of access to 3MTLs. i) Students’ perceptions on the purpose of 3MTLs. ii) Gadgets used by students to access 3MTLs. iii) Students’ perceptions on duration of 3MTLs

**Fig. 2:**
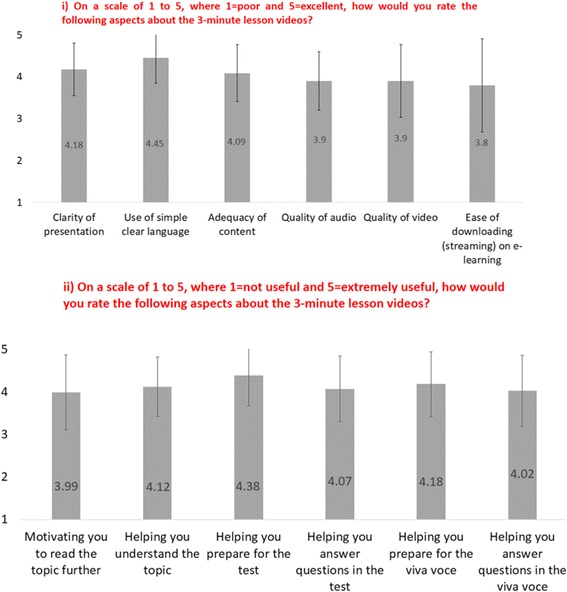
Quality and usefulness rating of 3MTLs. i) Student ratings on quality of 3MTLs on a 5-point Likert scale. ii) Student ratings on usefulness of 3MTLs on a 5-point Likert scale

For analysis of responses from the Part B questionnaire (for data from items B1 to B10, see Additional file 4: Table S3), students who indicated they did not use 3MTLs for either of the topics (*n* = 19) were excluded. The majority of responses indicated that students used 3MTLs for revision while, a smaller percentage also used it for orientation, and studying (Fig. 1i). Smartphones and laptops were the major gadgets used by students to access 3MTLs (Fig. 1ii). When questioned about their perception of the length of the podcast, 37 students (49.3%; Fig. 1iii) indicated that the time duration of podcasts was optimal, while an equal proportion felt that duration can be increased, preferably to 5 min. Mean scores on the rating of the quality of 3MTLs were lowest for *ease of downloading (streaming) on e-learning* and highest for the *use of simple, clear language* (Fig. 2). Mean scores for the usefulness of 3MTLs were lowest for *motivation obtained to the read the topic* and highest for *helping to prepare for the test* (Fig. 2). Also, an overwhelming majority (96%; *n* = 72) of students indicated that they would recommend 3MTLs for other topics in future.

**Fig. 3:**
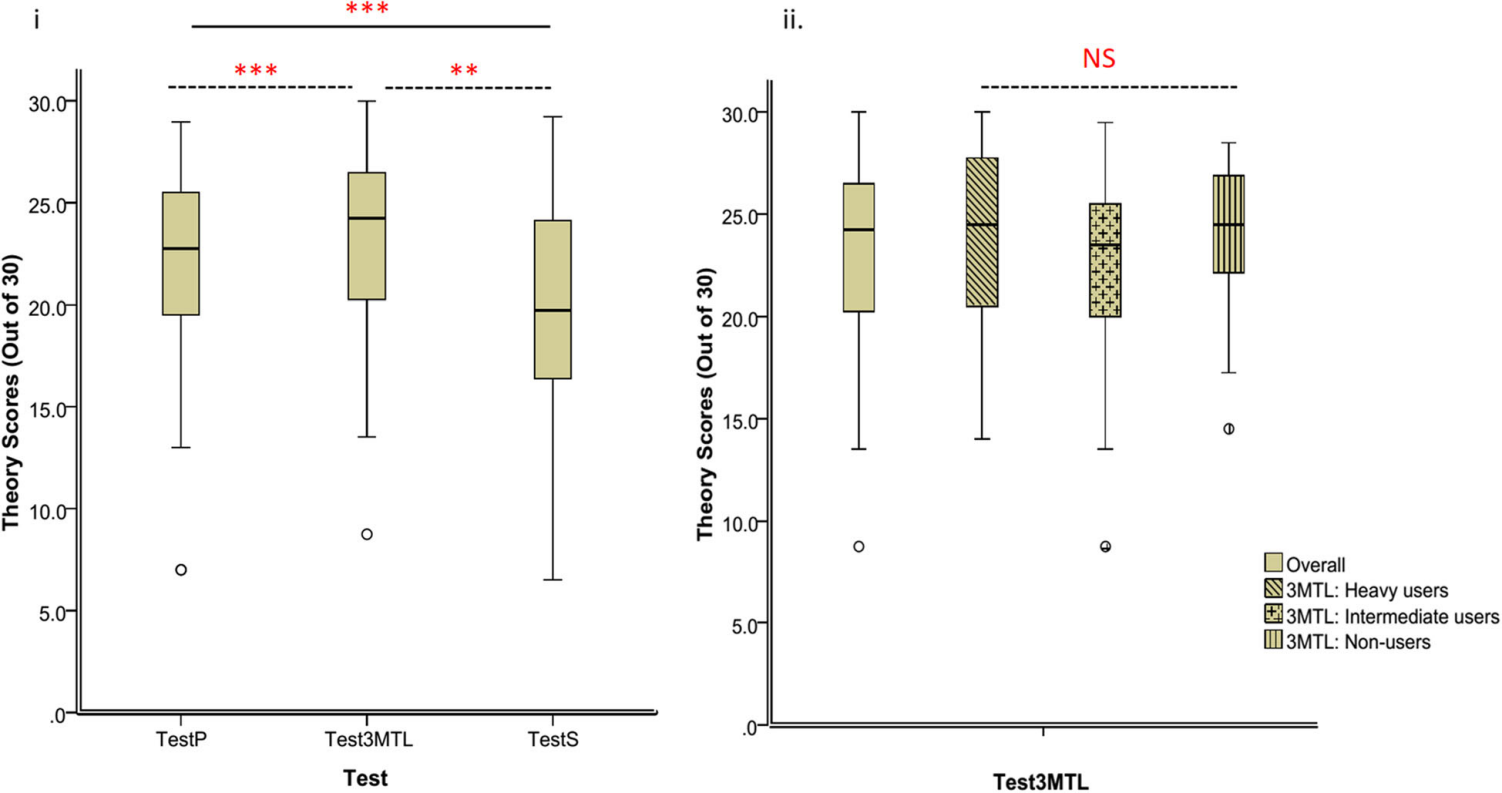
Comparison of scores of students in written assessments. i) Scores in the test (Test_3MTL_) which had implemented 3MTLs were compared with assessments that immediately preceded (Test_P_) and succeeded (Test_S_). Tests were scored out of a maximum of 30. Data is represented as mean ± SD in all groups (*N* = 92). **P < 0.01; ***P < 0.001. ii) Based on self-reported 3MTL usage, students were divided into 3 groups for comparison [3MTL: Heavy-users (used 3MTLs for two topics; *N* = 41); 3MTL: Intermediate-users (used 3MTLs for some of the topics; *N* = 32); 3MTL: Non-users (did not use 3MTLs; *N* = 19)]. Tests were scored out of a maximum of 30. Data is represented as mean ± SD in all groups. NS- Not significant

Overall, average scores in Test_3MTL_ were not different when compared with Test_P_ or Test_S_ or among student groups based on 3MTLs usage (Fig. 3). However, the difference of scores in pairwise comparisons (Test_3MTL_ vs. Test_P_; Test_3MTL_ vs. Test_S_) was statistically significant (Table 3). In the pairwise comparisons with students groups stratified based on 3MTL usage, the difference was significant amongst 3MTL: Heavy-users in Test_3MTL_ vs. Test_P_. In the comparison of Test_3MTL_ vs. Test_S,_ all three subgroups showed significant pairwise differences. Also, the magnitude of the score difference amongst 3MTL: Heavy-users in pairwise comparisons were higher than both Intermediate and Non-users (Table 5). Further, 3MTL: Heavy-users were sub-classified into tertiles based on their average scores in historical assessments and pairwise comparisons were repeated (Additional file 5: Table S4). Notably, only the Above-average performers among the 3MTL: Heavy-users had consistently higher pairwise scores in the Test_3MTL_ vs. Test_P_ and Test_3MTL_ vs. Test_S_ comparisons (Table 5). Other demographic characteristics were similar in the subgroups of 3MTL: Heavy-users (data not shown).

**Table 5:** Pairwise comparison of students’ performance in assessments

Groups	N	Paired differences				Significance (2-tailed)
		Mean	SEM	99% Confidence interval		
				Lower	Upper	
T_3MTL_ - T_P_						
Overall	94	1.67	0.43	0.53	2.8	**<0.001**
3MTL: Heavy-users	41	2.32	0.74	0.32	4.31	**0.003**
3MTL: Intermediate-users	34	1.07	0.67	−0.75	2.9	0.117
3MTL: Non-users	19	1.33	0.76	−0.86	3.52	0.098
T_3MTL_ - T_S_						
Overall	92	3.3	0.43	2.19	4.47	**<0.001**
3MTL: Heavy-users	41	4.20	0.66	2.43	5.97	**<0.001**
3MTL: Intermediate-users	32	2.63	0.73	0.62	4.64	**0.001**
3MTL: Non-users	19	2.63	0.91	0.003	5.26	**0.01**
Analysis of subgroups of 3MTL: Heavy-users						
T_3MTL_ - T_P_						
Below-average performers	13	2.94	1.74	−0.84	6.73	0.116
Average performers	14	2.57	1.38	−0.41	5.55	0.085
Above-average performers	14	1.48	0.58	0.24	2.73	**0.023**
T_3MTL_ - T_S_						
Below-average performers	13	6.40	1.07	4.06	8.75	**<0.001**
Average performers	14	4.66	1.33	1.78	7.54	**0.004**
Above-average performers	14	1.70	0.51	0.59	2.80	**0.005**

Paired differences of scores in the test (Test_3MTL_) which had implemented 3MTLs were compared with assessments that immediately preceded (Test_P_) and succeeded (Test_S_). Based on self-reported 3MTL usage, students were divided into 3 groups for comparison [3MTL: Heavy-users (used 3MTLs for two topics); 3MTL: Intermediate-users (used 3MTLs for some of the topics); 3MTL: Non-users (did not use 3MTLs]. For intragroup comparison, students were classified into tertiles based on their average scores in the historic assessments conducted prior to Test_P_ (Below-average performers, Average performers, and Above-average performers). All tests were scored out of a maximum of 30. SEM: Standard Error of Mean. Paired t-test was used for all comparisons. Significant *p* values are highlighted in bold

## Discussion

This pilot study was designed to analyze the perceptions of medical students to short-duration podcasts in biochemistry. Specifically, the study was designed to analyze how and why students accessed podcasts and their experiences in the process. Additionally, an exploratory analysis of the use of podcasts and students’ performance in the assessments was also done. Overall, podcasts were well received in this study and students perceived 3MTLs mainly as a supplementary learning tool. Students felt that 3MTLs were a valuable addition to routine teaching practice and suggested extending it to other topics. The results of the qualitative and quantitative analysis indicated that students mainly used 3MTLs for revision purpose after having read the topic. Particularly, students reported using 3MTLs for a quick review before assessments. This aspect was corroborated with a spike in the number of visitors and views of 3MTLs a day before assessment in the e-learning portal (data not shown). A smaller proportion of students used 3MTLs as a preparatory tool for gaining a topic overview. Additionally, students also felt that 3MTLs were helpful in improving their understanding of the topic, clarify concepts and focus on important points. These findings are consistent with other previous studies involving podcasts [5, 24, 30–32, 53].

Interestingly, half of the students perceived that the length of podcasts was optimum while an equal proportion suggested that the duration of podcasts can be increased to about 5 min. The survey by Matava et al. showed that podcast listeners preferred different podcast durations depending on the content [41]. For case presentations, procedural skills, journal article summaries, and discussions, the preferred podcast duration were 5–15 min, while for recorded lectures, a 15–30 min podcast was preferred; podcasts >45 min were least preferred. Matava et al. explain this observation as “respondents perceive that a certain amount of time is necessary to convey key learning objectives” [41].

Respondents in our study also indicated a preference to the audio and visual component in podcasts similar to few earlier observations [5, 34, 41]. Students noted that the teacher’s voice-over in the background was reassuring and useful in learning while the visual component in the podcasts made them lively. A few students felt that 3MTLs were more useful and interesting compared with reading texts. However, these observations were not shared by the majority, and a few students specified their preference to reading as the reason for not using 3MTLs. These observations further reiterate about different learning preferences among students. Certainly, the inclusion of audio-visual component in podcasts would be advantageous for students with aural, visual or multimodal learning preferences [2].

It was noted that the majority of students reported using smartphones to access 3MTLs followed by laptops. This observation was an interesting contrast since, several studies conducted before 2014 have reported that podcasts were accessed by listeners mainly through computers followed by portable media players [5, 6, 10, 31–33, 37, 41, 54–57]. However, general podcast listenership trends in the USA and UK has shown a shift towards smartphones after 2014 [58, 59]. Keeping in line with this trend, the more recent studies in medical education also indicate that podcasts are mainly accessed through smartphones over computers [60, 61] and our results corroborate with this observation.

Attention to technological issues of podcasts and their perceptions to users are important. Earlier observations show that technological issues are often cited by students as barriers to accessing podcasts [32]. Findings from this present study also indicate technical issues: ease of downloading (streaming) 3MTLs had lower ratings (Fig. 2 ii) and several students who did not access podcasts cited download issues and bigger file size as reasons. Additionally, the ease of downloading was rated lower among students who accessed 3MTLs on phones compared to computers (data not shown). A probable reason for this could be that podcasts were made available through the institution’s e-learning portal, a website best suited for use with computers. An alternative option to overcoming issues with the medium of delivery would be to make podcasts available through more than one portal that allows video streaming (e.g., YouTube, Facebook). Other device-related technical factors could be issues related to internet connectivity, server, browser compatibility, device’s storage capacity, software or hardware. Concerning the format of podcasts, the major feedback provided by students in this study was towards the improvement of audio and video quality. A major reason could be that only basic equipment was used for recording the podcasts. The investigator’s lack of experience in podcasting could be another contributing factor. Also, 3MTLs were made available in 320 × 240 image resolution ideally suited for viewing in handheld gadgets. It is possible that students who accessed podcasts on devices with larger screens may have rated the video quality lower. However, production of high-quality video podcasts would also require more preparation time, costlier recording equipment, and larger file sizes, amongst other factors. The costs and benefits would need to be weighed while considering the production of video podcasts. However, it is important for podcasters to be aware of these issues while deciding on the format and medium of delivery of podcasts.

An exploratory comparison of scores in summative assessments also suggested that use of 3MTLs was associated with better performance. A few studies in the past have used a pre-test-post-test quiz to assess the effectiveness of podcast implementation in comparison to lecture-teaching and showed weak evidence for improvement in podcast group [25–27]. Comparison between teaching with lectures and video podcasts by a crossover trial observed that student evaluations were not different between the two formats [8]. In this study, the overall mean scores were not different between students based on 3MTLs usage, suggesting that factual recall is similar amongst students, irrespective of podcast usage. However, the pairwise comparisons revealed better scores amongst 3MTL: Heavy users. It is further possible that a subset of students in 3MTL: Heavy-users may be the primary beneficiaries of 3MTLs since sub-stratification of 3MTL: Heavy users showed that benefit was consistently observed only amongst ‘Above-average performers.’

Some potential confounders of this study are factors about the student’s background related to school education, study habits, learning preferences, and availability of gadgets to access 3MTLs (Additional file 6). The questionnaire was designed to collect information on some of the above factors, but, no patterns were detected in the use of 3MTLs (data not shown). Participants were recruited by convenience sampling, and hence there is a possible sampling bias in the study. There is a possibility of response bias considering that investigator (AR) and participants are well acquainted and AR was also involved in the delivery of lectures, creation on 3MTLs and feedback collection. Another limitation of this study would be the lack of baseline pre-test scores on the topics under study. Finally, data on the total number of views and visitors from the website statistics may be an underestimate, since it does not account for files downloaded and transferred to students by other means. In comparison to previous studies, there are also a few aspects of the methodology of this current study that are worth mention. A major difference would be that fixed, short-duration podcasts aimed at providing highlights of the topic were used in this study, while others have used moderate- or long-duration podcasts [8, 25, 30, 34]. Also, in this study 3MTLs were only provided as a supplementary tool, and hence, assessments were held as per routine teaching schedule, and no additional assessments (specifically related to 3MTLs) were conducted.

### Authors’ perspectives

Informal feedback from medical students at CMC, Vellore often suggests that students find biochemistry a difficult subject to study and remember. Lecture capture is not practiced in the author’s institution, and hence students are limited to, lectures and text-based learning resources. The idea for developing podcasts evolved to provide additional resources for students with different learning preferences. The inspiration of the time frame was partly from the Three Minute Thesis competition conducted by University of Queensland, Australia (https://threeminutethesis.uq.edu.au/). The responses from faculty and students for pilot podcast episode were motivating, and the idea eventually evolved to cover the entire module. The author’s department and institution were supportive, however, no additional resources were obtained from any source. The podcast recording and delivery were done by the author himself using his personal equipment. Planning and creation of each podcast episode required about 6–8 h. The script for the podcast was written in advance and rehearsed multiple times to ensure it fits the time frame. The author found the implementation of podcasts in his teaching module was, by itself, an enriching experience. Though podcast development is laborious, they can be reusable tools to deliver high-yield information to students and may also partly relieve the burden on faculty occupied with clinical, research or administrative responsibilities. In institutions, that do not follow lecture-capture approach, short and moderate-duration podcasts can be useful alternatives.

## Conclusion

To conclude, short-duration podcasts are useful supplementary tools in medical education. Analysis of the feedback from students suggests that they consider podcasts as a very useful tool for review and preparation for assessments. The authors’ beliefs about the future of this media concur with others who view podcasts as a supplementary learning tool but not meant to replace traditional teaching.

## Additional files

Additional file 1: Part B – Questionnaire. (DOCX 23 kb)

Additional file 2: Table S1. Students' responses to question A1. (DOCX 13 kb)

Additional file 3: Table S2. Students' responses to question A2. (DOCX 17 kb)

Additional file 4: Table S3. Data from responses for items B3-B10. (DOCX 15 kb)

Additional file 5: Table S4. List of assessment topics. (DOCX 12 kb)

Additional file 6: Table S5. Average scores of 3MTL heavy users in assessments. (DOCX 12 kb)

## Abbreviations

3MTL: 3-minute lesson
SRQR: Standards for Reporting of Qualitative Research
