# Supplementary material for: Short-duration podcasts as a supplementary learning tool: perceptions of medical students and impact on assessment performance
Source: BMC Med Educ. 2017 Sep 18;17:167. doi: 10.1186/s12909-017-1001-5 (PMC5604391; doi:10.1186/s12909-017-1001-5)
Supplement: Supplementary file 1 — Part B – Questionnaire. (DOCX 23 kb) [file 12909_2017_1001_MOESM1_ESM.docx]

**Name: Age: Gender:**

1. **Board of studies for Class XII:**

a) State board b) CBSE c) ICSE d) Others, please specify:

1. **Year of passing class XII:**
2. **Medium of education for Class X:** a) English b) Regional language
3. **Medium of education for Class XII:** a) English b) Regional language
4. **On an average, how many hours per week do you spend on studying Biochemistry?**

a) <2 hours (15 min a day) b) 2-4 hours (30 min a day) c) >4 hours (30 min to 1 hr a day)

d) Only before tests e) Others, please specify:

1. **How would you describe your study habits?**

a) I prefer studying alone b) I often study with a friend/friends (2-3)

c) I prefer to study in groups (3 or more) d) Others, please specify:

1. **Which of the following places do you prefer for your study? (Indicate all that apply)**

a) Hostel b) Library c) College campus d) Others, please specify:

1. **Which of the following learning resources do you access for Biochemistry? (Indicate all that apply)**

a) Lecture slides b) Textbooks c) E-books d) Internet sources

e) Online videos f) Others, please specify:

1. **Did you use 3-minute lessons to study for the topic- fat soluble vitamins?**

a) Yes b) No

1. **Did you use 3-minute lessons to study for the topic- heme metabolism and disorders of hemoglobin?**

a) Yes b) No

1. **What was the purpose behind your using 3-minute lesson videos? (Indicate all that apply)**

a) To get orientated to the topic before studying it in detail

b) To review the topic after studying it

c) To study the topic quickly since you did not have much time before the test

d) Others, please specify:

1. **How did you access the 3-minute lesson videos? (Indicate all that apply)**

a) Smartphone b) Tablet c) Laptop d) Desktop e) Others, please specify:

1. **Which of the following statements on the time duration of the 3-minute lessons is agreeable to you?**

a) 3 minutes is less. Time duration of the videos can be increased to ______ minutes

b) Time duration of the videos is optimum, I wouldn’t suggest changing it

c) 3 minutes is long. Time duration of the videos can be shortened to ______ minutes

1. **On a scale of 1 to 5 (where 1=poor, 2= fair, 3=good, 4=very good and 5=excellent), how would you rate the following aspects about the 3-minute lesson videos?**

|  | Rating |
| --- | --- |
| Clarity of presentation |  |
| Use of simple clear language |  |
| Adequacy of content |  |
| Quality of audio |  |
| Quality of video |  |
| Ease of downloading (or streaming) on e-learning |  |

1. **On a scale of 1 to 5 (where 1=not useful, 2=somewhat useful, 3=useful, 4=very useful and 5=extremely useful), how would you rate the following aspects about the 3-minute lesson videos?**

|  | Rating |
| --- | --- |
| Motivating you to read the topic further |  |
| Helping you understand the topic |  |
| Helping you prepare for the test |  |
| Helping you answer questions in the test |  |
| Helping you prepare for the viva voce |  |
| Helping you answer questions in the viva voce |  |

1. **Would you recommend 3-minute lesson videos for other topics in the future?** a) Yes b) No
