# Supplementary material for: Short-duration podcasts as a supplementary learning tool: perceptions of medical students and impact on assessment performance
Source: BMC Med Educ. 2017 Sep 18;17:167. doi: 10.1186/s12909-017-1001-5 (PMC5604391; doi:10.1186/s12909-017-1001-5)
Supplement: Supplementary file 2 — Students' responses to question A1. (DOCX 13 kb) [file 12909_2017_1001_MOESM2_ESM.docx]

| Item | Response options | Responses (%) |
| --- | --- | --- |
| Gender | Male | 42.6 |
|  | Female | 57.4 |
| B3. Medium of education in class X | English | 97.9 |
|  | Regional language | 2.1 |
| B4. Medium of education in class X | English | 98.9 |
|  | Regional language | 1.1 |
| B5. On an average, how many hours per week do you spend on studying Biochemistry? | | |
|  | <2 hours (15 min a day) | 4.3 |
|  | 2-4 hours (30 min a day) | 13.8 |
|  | >4 hours (30 min to 1 hr a day) | 4.3 |
|  | Only before tests | 69.1 |
|  | Others | 6.4 |
|  | No response | 2.1 |
| B6. How would you describe your study habits? | | |
|  | I prefer studying alone | 61.7 |
|  | I often study with a friend/friends (2-3) | 25.5 |
|  | I prefer to study in groups (3 or more) | 1.1 |
|  | Others | 8.4 |
|  | No response | 3.2 |
| B7. Which of the following places do you prefer for your study? | | |
|  | Hostel | 36.2 |
|  | Library | 21.3 |
|  | College campus | 4.3 |
|  | Others | 12.4 |
|  | Hostel/library | 18.1 |
|  | Hostel/college | 5.3 |
|  | No response | 1.1 |
| B8. Which of the following learning resources do you access for Biochemistry? | | |
|  | Slides | 95.7 |
|  | Textbooks | 86.2 |
|  | E-books | 25.5 |
|  | Internet sources | 23.4 |
|  | Online videos | 31.9 |
| B9. Did you use 3-minute lessons to study for the topic- fat soluble vitamins? | | |
|  | Yes | 69.1 |
|  | No | 30.9 |
| B10. Did you use 3-minute lessons to study for the topic- heme metabolism and disorders of hemoglobin? | | |
|  | Yes | 54.3 |
|  | No | 45.7 |

**Additional table 2. Student responses to item A1.**
