# Supplementary material for: Short-duration podcasts as a supplementary learning tool: perceptions of medical students and impact on assessment performance
Source: BMC Med Educ. 2017 Sep 18;17:167. doi: 10.1186/s12909-017-1001-5 (PMC5604391; doi:10.1186/s12909-017-1001-5)
Supplement: Supplementary file 3 — Students' responses to question A2. (DOCX 17 kb) [file 12909_2017_1001_MOESM3_ESM.docx]

| Item **A1. Did you find the 3-minute videos helpful? Please elaborate** | |
| --- | --- |
| **S. No** | **Students’ comments** |
| 1 | *The lectures are good as they provide plenty of information in a short time interval. Good for revision.* |
| 2 | *The 3-minute lesson was a brilliant idea. It acts as a last minute revision tool for exam. It was very useful for me. Although I prefer reading from book* |
| 3 | *Yes. Good for quick revision* |
| 4 | *Yes, they are of real use during last minute revision before exams.* |
| 5 | *Yes, such lectures are useful for revision before viva and get good marks.* |
| 6 | *Yes. It was helpful in revision of classes after completing the portion. I watched them on the morning before the test and found it very concise and beneficial.* |
| 7 | *Yes it was useful for last minute revision.* |
| 8 | *Yes I found it useful because it helped for quick revision before viva and exams. Proved less boring than reading through texts and slides.* |
| 9 | *Yes. Very useful for last minute revision. Summarizes the whole chapter. Easy to understand.* |
| 10 | *Found it really useful. It was really awesome. It was really helpful to revise the topic in the last minute* |
| 11 | *It was very helpful. Especially for revision as it was short and easy to understand. It helped me understand better after I have gone through the slides once or twice.* |
| 12 | *Yes, they were useful. Once after going through the notes the video lectures were like revision (summarized) on the go. So instead of listening to music sometimes you could listen to them for a fresh recap of memory.* |
| 13 | *Yes very very useful.* |
| 14 | *Yes, it was very useful. It helped me a lot during revision session.* |
| 15 | *Yes, good review session before exams.* |
| 16 | *Yes it was helpful during revision.* |
| 17 | *Yes, the 3 minute videos were very useful to revise and recollect the points we've learnt in class. It also helps me to get the personal touch as the teacher himself is speaking to us.* |
| 18 | *Yes. Pretty much. It helped us to revise important topics at the last moment where there is insufficient time to revise everything.* |
| 19 | *Yes, it was useful to have a concise form of the lectures. It was especially useful for revising before the exam.* |
| 20 | *Yes. It was very useful in having a better understanding of the topic and it helps you to remember the concepts better.* |
| 21 | *Yes, the videos were useful especially when viva was there for a quick revision. Additional point is that it helps you to understand the layout of the slide.* |
| 22 | *Very nice. Very useful in revision before the exam.* |
| 23 | *Yes, it really helped me revise it was really good to refresh memory.* |
| 24 | *Yes they are useful to recap. Thank you sir for putting effort and going the extra mile for us.* |
| 25 | *Yes, they were perfect, adequate and to the point.* |
| 26 | *Yes, helps in understanding concepts day before exam.* |
| 27 | *Wonderful tool for revision. Also for good overview before studying.* |
| 28 | *Yes it is useful for revision just before the exam/viva.* |
| 29 | *Yes I found it useful. I revised by watching the videos on the morning of the exam.* |
| 30 | *Yes. If we are studying from slides it helps to understand it better. No doubts.* |
| 31 | *Yes it was nice.* |
| 32 | *It was a brilliant and innovative idea. It was very useful for revising after I completed the portion.* |
| 33 | *The lessons were very useful. Especially heme metabolism before test.* |
| 34 | *Yes, because after completely studying about a vitamin watching the video made it easier to recollect everything once again before going to the exam. Anything which I felt that I had a doubt I could read once again.* |
| 35 | *Yes, it was pretty innovative in the sense that it is like last minute revision just before exam, to make sure that one has covered everything up before a test.* |
| 36 | *Yes, very useful. It very much helps in studying and revising at last.* |
| 37 | *Yes, before I started reading the topic I referred to the videos which gave me a head start to what I was supposed to know.* |
| 38 | *Yes, these videos are very useful for quick revision before exams. Thank you sir for your efforts.* |
| 39 | *Very useful for quick revision - runs through the slides fast so you can recollect points.* |
| 40 | *Useful for me. I revise topic in 3 minutes and it is good for viva preparation.* |
| 41 | *Yes, much better way to recapitulate especially with teacher’s voice in the background.* |
| 42 | *Yes it was interesting and was much easier than going through the e-learning slides.* |
| 43 | *Yes. Audiovisual aids can be more useful than just reading a book for some students. Useful as a quick revision.* |
| 44 | *Yes. I found it to be very useful. Somehow listening to an hour lecture and the 3 min video lecture where whole topic was briefed made no difference in a sense that it covered all the main facts to be known. It's a good starter to the topic we're going to read.* |
| 45 | *Yes sir. It is a very useful tool to revise what I studied from slides. I could also catch up things I missed by merely reading the slides. I could hear a teacher speaking and it was really useful. If I cannot finish studying slides just before test I can quickly see the video.* |
| 46 | *Yes, very useful. At least for me after reading the topic before exam when I wanted to revise the heading I used the video to do so.* |
| 47 | *Helpful for quick overview.* |
| 48 | *Yes, they were very useful, especially for last minute revisions.* |
| 49 | *Yes they were useful. Especially for last minute revision.* |
| 50 | *Yes it was useful because at the end of learning the slides we could just revise and make sure that we still have in mind what we studied.* |
| 51 | *Yes it was easy for revision like I don't need to go through the slides again. I was able to revise whenever and wherever I want.* |
| 52 | *It was very useful as we got a short overview of our class. I learn better if a teacher explains stuff instead of reading books. I found it to be very innovative.* |
| 53 | *Very useful to revise the topic on the morning of the exam.* |
| 54 | *Useful. Compressed info in short. Apt for the topic, don't think it would be good for pathways etc.* |
| 55 | *Yes. It is a good way to revise the subject as everything important condensed into 3 minutes.* |
| 56 | *The videos were useful. It helped to a last minute revision and recollect the content.* |
| 57 | *Yes. They were very useful because it helped to revise topics, focus on important points. Convenient to study with phones.* |
| 58 | *I really loved the 3 minute lesson. I watched the videos on Monday. It helped me do a quick revision. I understood some things from a different perspective. And when I did watch the videos, I was really dying to tell you how much the lesson was useful and I thank you personally for it.* |
| 59 | *Yes. Very useful but it all depends on how well you knew the topic. Since it’s a pretty fast lecture I had to somewhat thorough with the topic before I listened to it. It's mainly only for revision I feel.* |
| 60 | *Yes, the videos were useful for revision.* |
| 61 | *Yes. It was very useful because it summarizes everything related to the topic. It is a good way to revise just before a test as there is hardly any time to study just 1 day before the test. It helps a lot in revising the day before test.* |
| 62 | *Yes. It was very helpful especially for last minute preparation and vivas. We were able to pause it and listen when we wanted and it was easy to understand the important concept rather than memorizing the stuff on slide which was hard to understand.* |
| 63 | *Yes. It was very useful for last minute preparation and also for revision. Helped remember things better.* |
| 64 | *Yes it was very useful. I even showed the videos to my friends who are studying in other medical colleges and they also found it useful and appreciated Dr. Anand very much.* |
| 65 | *Yes, they are useful as they gave a summary of what was explained in the whole presentation without taking much time.* |
| 66 | *Yes. The 3-minute lesson videos were very useful to study just the day before the exam. If possible make it available for all chapters.* |
| 67 | *Yes. It helped in understanding the categorization of specific topics and also to know the subheadings to answer a related question in the test (almost helped). It also helped in last minute revision for viva.* |
| 68 | *Yes. It was very useful for revision purpose after studying the subject. It was also helpful during viva to quickly recap everything in short time.* |
| 69 | *Yes. They are a great source of revising what you have learnt. I heard quite a few of them walking to the exam hall and I could write all those topics very well (or so I think).* |
| 70 | *Yes. They are useful for a last minute preparation or as a quick review.* |
| 71 | *Yes. It was very useful to go through once before studying - so we actually get an overview of it and even after studying it just as a revision. And because it's only 3 minutes it doesn't take much of our time and is very helpful. Especially with a teacher interacting unlike a normal ppt with just audio (which is super boring) the teacher's interaction through the video makes it even more lively.* |
| 72 | *Of course it was useful. In the future it will be more useful, I don't know about the past. The hi was reassuring and fantastic.* |
| 73 | *Very useful as a tool for quick revision and read before the exams. Easier to remember than lines in a text book.* |
| 74 | *Helpful for quick overview.* |
| 75 | *Yes, personally. I did not watch all the videos as I felt that the slides had adequate information and were easy enough to understand. But the videos are definitely useful and helpful in quick revision.* |
| 76 | *It is a good effort. But it is not downloadable. Please make it easier to download.* |
| 77 | *Somewhat, yes.* |
| 78 | *Useful only if we know the topic first. Like for revision.* |
| 79 | Yes, they were useful. But sir, considering the amount of time you put into making one video it is kind of unnecessary. It's like a substitution for your lecture - maybe beneficial for absentees to know what points were taught. But after attending lectures and taking class notes the videos are not absolutely essential. However they are nice, concise and clear concepts. |
| 80 | Sorry sir. I didn't use the videos. Studied with slides, easier to download. But I believe it may be helpful before university when there is lots of stuff to study |
| 81 | Sorry I didn't watch it. But the one I watched in class was useful. |
| 82 | Sorry I didn't watch any of the videos except the one sir showed in class. I think it will be useful to get points in 3 min in a capsule. |
| 83 | The video could been improved a lot. Animations could have been added. |
| 84 | Sorry sir, I did not watch the videos but my friend watched it and told it was very nice. During the class you showed one video and it was nice. |
| 85 | Sir I tried to download but I couldn't download. It had some problem with my phone. |
| 86 | Couldn't access the videos it requires a software to access on mobile. I have seen video in class though and it is extremely useful for revision. |
| 87 | Personally I didn't even watch a single video. I'm sorry for that because I prefer to study. I prefer reading. It was helpful for many students when I asked them. |
| 88 | I didn't watch that many but the ones I did watch were useful because it felt like I was listening through the lecture again and helped clarify a few doubts. |
| 89 | I actually haven't seen the video for my preparation before the test. But I do remember the video you showed in class and I think that it will be useful for those who have missed the class and for me too when I feel like listening something rather than reading it. |
| 90 | I tried my best to watch the videos but I failed in even watching them. Dear sir, please put up the videos for the next topic, I will definitely watch it. |
| 91 | No. Because it just seemed like you're reading out the slides, which I can do by myself. But it is useful for a quick revision if I already studied it before. |

Additional table 2. Student responses to item A1.
