# Supplementary material for: Short-duration podcasts as a supplementary learning tool: perceptions of medical students and impact on assessment performance
Source: BMC Med Educ. 2017 Sep 18;17:167. doi: 10.1186/s12909-017-1001-5 (PMC5604391; doi:10.1186/s12909-017-1001-5)
Supplement: Supplementary file 4 — Data from responses for items B3-B10. (DOCX 15 kb) [file 12909_2017_1001_MOESM4_ESM.docx]

| **Item A2. Do you have any suggestions to improve the videos?** | |
| --- | --- |
| **S. No** | **Students’ comments** |
| 1 | The voice could be a bit louder and slower in pace. |
| 2 | In the 3 minute video it was very full. Just your face and you were reading the whole thing. Apart from that you could have tried some jokes. |
| 3 | Use more colors and graphics. I hate white background. Don't sit and say. Stand change expressions. Good job, sir. I respect you for all the efforts you take for us. |
| 4 | Not every concept in ppt was covered in the 3 minute lesson. I think this happened because of the 3 minute time barrier. Instead you may use 4-5 minutes and explain all concepts. |
| 5 | Use of pictures related to clinical conditions will be helpful. |
| 6 | Don’t see any need to do them better. They are as concise as they can be. Good job, sir. |
| 7 | The 3 minute lesson video is nice but it would be more useful if it is more explanatory. And the video can be used to explain complex topics which students tend to forget so that we can hear your lesson on that topic again. |
| 8 | Instead of using same sentences in the slides it could be better if you can use your own language and explain. |
| 9 | Make it easier to download. |
| 10 | Encourage to continue it for future batches. Animations and background music required. |
| 11 | Video and sound quality. |
| 12 | Very nice. Keep it up. |
| 13 | You can make videos for every concept in biochemistry if you can and have come because you mentioned that each video is taking 6 hours. But it's a great move. |
| 14 | Sound quality. |
| 15 | Expand to other topics as well. |
| 16 | Can you please cover other important topics from different chapters. |
| 17 | I hope this is extended for the lectures. Good effort. |
| 18 | It would be better if the video had a synopsis on what are the topics that are covered. |
| 19 | Nothing. Thank you so much sir for spending so much of your time for us. |
| 20 | Include all data from the ppt slide if possible. |
| 21 | Could you make the presentations a little more elaborate as in explaining stuff a bit more may be making it 5 minutes or so. |
| 22 | Better audio quality. Longer videos with more concepts. |
| 23 | Increase the minutes. |
| 24 | A little more elaborate a minute longer video would have been better. |
| 25 | Put more concepts in it. |
| 26 | Projecting the speaker's face is not deemed to be that important, considering that one can save much time in preparation. |
| 27 | If there's more stress and focus on the questions asked in viva or important topics to deal in that chapter. |
| 28 | They are very nice. I found it completely fine, no need it to improve. |
| 29 | Extra information apart from that on the slides should be added to the presentations. |
| 30 | I liked the hi. It is very good. |
| 31 | Sound quality. Pace is fast and make it downloadable. |
| 32 | It could be merged with slides. Sometimes it is difficult to download. |
| 33 | We cannot download the videos. Please make it possible to download. |
| 34 | Improvement in audio quality. 5 minute lesson videos to explain more concepts. |
| 35 | No suggestions. |
| 36 | Stress a little more on important points. |
| 37 | No suggestions. Sir you are awesome. |
| 38 | Putting animations. |
| 39 | No suggestions. |
| 40 | It's good. I don't have any particular suggestions to improve it. |
| 41 | Make it 5 minute please. |
| 42 | Have it played before class rather than after. |
| 43 | Bacground music. Small file size so that it is easy downloadable. |
| 44 | No suggestions. |
| 45 | Nothing. |
| 46 | Voice-clearer. |
| 47 | No suggestions. |
| 48 | Improve resolution of videos. |
| 49 | I love the fact that its 3 min short stuff is always cool. |
| 50 | Please do it for the big lectures as in carbs, proteins etc. |
| 51 | Please mention questions that may come from the topic. |
| 52 | Better mic quality. 1 minute extra for clinical importance. |
| 53 | No suggestions. |
| 54 | Should make more such videos. Please pay it in class too as lectures are so boring. |
| 55 | The videos could be a little more interactive. |
| 56 | Good. |
| 57 | 3-minutes is very less so better make it 5 minutes. It would be better if you put up another 3 minute video for renal function tests topic. Really in need of a better and clear explanation. |
| 58 | All the subheadings that is necessary for the written answer in a test could be mentioned. The lecture can be taken in a flow/order easier to know how to answer the question. |
| 59 | To touch through more headings reducing the explanation for each. |
| 60 | Since it was vitamins and porphyria, the topics were short. If you plan to make it for all the topics I don't know how it'll work for proteins and carbs. But they are really good. |
| 61 | Please smile a little while talking sir. Extra 3 minute videos with a few extra information could also be uploaded so ppl interested can use that too. |
| 62 | Extend the video to 5 minutes and include more information. Better sound systems could be helpful. |
| 63 | A list of important questions from the covered topic can be inserted at the end of every video. |
| 64 | Serves the purpose. Would be better if presentations purely based on clinical relevance are made. |
| 65 | Really good job sir. Innovative. |
| 66 | Enhanced technology will do. More clarity and related stuff. Rest it was too good. Keep going. |
| 67 | The time for video could have been taken for at least 10 minutes, properly describing in detail could be helpful. The video should be implemented in class in the absence of lecturer. |
| 68 | Clarity needed. |
| 69 | If it is possible change the format of the video or if it is uploaded on YouTube. |
| 70 | The videos took some time to download and take up a lot of memory. However, they helped a lot before the test for revision. |
| 71 | Maybe explain the most important topics a little more. However, the shortness of the videos is what makes them interesting. |
| 72 | If you had time it would be better if small videos with moving figures are made instead of just explaining information. Maybe this can be used in case of concepts. |
| 73 | May be sir, you can make videos on topics you did not teach (carbohydrates, proteins) so that we understand them better. We have a lot of uncleared doubts in those topics. |
| 74 | Speak loudly, clearly and slowly. |
| 75 | Maybe if you add new interesting content related or ask viva questions. But that wouldn't serve for quick revision. So, depends on your objective for making the videos. Revision? Sparking interest? I'd prefer sparking interest. |
| 76 | Voice clarity not so great. |

Additional Table 3. Student responses to item A2.
