# Supplementary material for: Short-duration podcasts as a supplementary learning tool: perceptions of medical students and impact on assessment performance
Source: BMC Med Educ. 2017 Sep 18;17:167. doi: 10.1186/s12909-017-1001-5 (PMC5604391; doi:10.1186/s12909-017-1001-5)
Supplement: Supplementary file 5 — List of assessment topics. (DOCX 12 kb) [file 12909_2017_1001_MOESM5_ESM.docx]

| **Assessment** | **Topics of modules covered in assessment** |
| --- | --- |
| Historic assessments | |
| Test 1 | Cell, Structure and functions of proteins, Enzymes |
| Test 2 | Chemistry and metabolism of carbohydrates |
| Test 3 | Chemistry and metabolism of proteins |
| Test 4 | Chemistry and metabolism of lipids, Integration of metabolism |
| Test 5 | Bilirubin metabolism, Liver function tests and jaundice |
|  | |
| Test_P_ | Chemistry and metabolism of nucleotides |
| Test_3MTL_ | Fat soluble vitamins, Heme metabolism and disorders of hemoglobin |
| Test_S_ | Water soluble vitamins and biological oxidation |

Additional table 4. List of assessment topics
