# Supplementary material for: Short-duration podcasts as a supplementary learning tool: perceptions of medical students and impact on assessment performance
Source: BMC Med Educ. 2017 Sep 18;17:167. doi: 10.1186/s12909-017-1001-5 (PMC5604391; doi:10.1186/s12909-017-1001-5)
Supplement: Supplementary file 6 — Average scores of 3MTL heavy users in assessments. (DOCX 12 kb) [file 12909_2017_1001_MOESM6_ESM.docx]

**Additional table 5. Average scores of 3MTL heavy users in assessments**

| **Additional table 5. Average scores of 3MTL heavy users in assessments** | | | | | |
| --- | --- | --- | --- | --- | --- |
| **Subgroups of 3MTL: Heavy users** | **N** | **Average in historic assessments**  Mean (SD) | **Test_P_**  Mean (SD) | **Test_3MTL_**  Mean (SD) | **Test_S_**  Mean (SD) |
| Below-average performers | 13 | 12.3 (2.2) | 17.3 (4.3) | 20.3 (3.9) | 13.8 (4.1) |
| Average performers | 14 | 16.8 (1.1) | 21.5 (3.8) | 24.1 (3.0) | 19.4 (4.3) |
| Above-average performers | 14 | 23.0 (2.2) | 25.2 (2.6) | 26.7 (3.0) | 25.0 (3.8) |
